# Population Pharmacokinetics of Tralokinumab in Adult Subjects With Moderate to Severe Atopic Dermatitis

**DOI:** 10.1002/cpdd.1113

**Published:** 2022-06-07

**Authors:** Anders Soehoel, Malte Selch Larsen, Stine Timmermann

**Affiliations:** ^1^ Clinical Pharmacology LEO Pharma A/S Ballerup Denmark; ^2^ Present address: Novo Nordisk Søborg Denmark

**Keywords:** atopic dermatitis, IL‐13, population pharmacokinetics, tralokinumab

## Abstract

Tralokinumab is the first biologic therapy for moderate‐to‐severe atopic dermatitis (AD) that specifically neutralizes interleukin‐13 activity, a key driver of AD signs and symptoms. Tralokinumab is a human immunoglobulin G4 monoclonal antibody administered subcutaneously every 2 weeks (with possibility of maintenance dosing every 4 weeks). This population pharmacokinetic (PK) analysis aimed to identify sources of PK variability and relevant predictors of tralokinumab exposure in adults with moderate to severe AD. Nonlinear mixed‐effect modeling, including covariate analysis, was used on a data set including 2561 subjects (AD, asthma, healthy) from 10 clinical trials. A 2‐compartment model with first‐order absorption and elimination adequately described the tralokinumab PK. Body weight was identified as a relevant predictor of tralokinumab exposure; other covariates including age, sex, race, ethnicity, disease type, AD severity, and renal and hepatic impairment were not. For body weight, the difference in exposure between the upper‐ and lower‐weight quartiles in patients with AD was <2‐fold, supporting the appropriateness of flat dosing (300 mg). Given the reduced exposure associated with higher body weight, coupled with the reduced exposure provided by dosing every 4 weeks, it is uncertain whether higher‐weight patients will achieve sufficient exposure to maintain efficacy if dosed every 4 weeks instead of the standard every 2 weeks.

Atopic dermatitis (AD) is the most common inflammatory skin disease in the developed world.^1^ In a web‐based survey using a modified UK Working Party definition of AD, the point prevalence of AD in adults ranged from 2.1% to 8.1% across individual countries in Europe, North America, and Japan.^2^ AD is associated with a significant disease burden and impact on mental health, quality of life, and lifestyle.^3^ Symptoms in moderate to severe AD include intense itch, xerosis, and recurrent eczematous skin lesions.^1^

The immunoregulatory cytokine interleukin (IL)‐13 plays a key role in the pathogenesis of AD, amplifying the inflammatory response and skin barrier disruption, activating itch signaling, and increasing the risk of skin infections.^4^, ^5^, ^6^ The complex interplay of these effects leads to a vicious cycle of further immune activation, and the overexpression of IL‐13 in AD skin correlates with disease severity.^7^, ^8^

The pharmacological treatment for AD progresses from mild to high‐potency topical anti‐inflammatory therapy and in many cases of moderate to severe AD to systemic immunomodulatory therapy.^9^, ^10^, ^11^ Currently, 2 biologic therapies are available for the treatment of moderate‐to‐severe AD: dupilumab—an IL‐4 receptor α antibody that blocks IL‐4 and IL‐13 signaling—and tralokinumab.

Tralokinumab is a fully human immunoglobulin G4 monoclonal antibody that binds to IL‐13 with high affinity at an epitope that overlaps with the binding site of the IL‐13Rα1 receptor, preventing IL‐13 from binding to IL‐13Rα1.^12^, ^13^, ^14^, ^15^ Tralokinumab is developed to specifically neutralize IL‐13, a key driver of AD signs and symptoms.^4^, ^15^, ^16^ The drug provides significant and early improvements in signs and symptoms of moderate‐to‐severe AD^17^ and normalizes the expression of several AD biomarkers.^18^ Tralokinumab is approved in several regions and countries for the treatment of moderate to severe AD in adult patients who are candidates for systemic therapy. The drug is administered by subcutaneous (SC) injection, and the recommended dose in adults is an initial dose of 600 mg followed by 300 mg administered every 2 weeks. Dosing every 4 weeks may be considered for patients who achieve clear or almost clear skin after 16 weeks of treatment with the every‐2‐weeks regimen, although reducing the dosage to every 4 weeks might not be appropriate for patients with higher body weight (>100 kg). Tralokinumab is currently under development for the treatment of moderate to severe AD in adolescents (NCT03526861).

During an earlier, discontinued development program for tralokinumab in asthma, a population pharmacokinetic (PK) analysis of tralokinumab, using data from both adults and adolescents, was used to evaluate the optimal dosing strategy for adolescent subjects with asthma.^19^ The results indicated that tralokinumab has linear PK, with no evidence of target‐mediated drug disposition. This previous analysis did not include subjects with AD. We have now analyzed a substantially larger data set comprising data from phase 1 to phase 3 trials, including subjects with AD in addition to subjects with asthma and healthy subjects, resulting in a thorough evaluation of the influence of various covariates on the exposure of tralokinumab in subjects with AD.

The purpose of this analysis is to describe the PK of tralokinumab in subjects with moderate to severe AD. The analysis is aimed at identifying sources of variability in the population and, in particular, relevant predictors of tralokinumab exposure that could inform decisions about the clinical use of tralokinumab, including consideration of weight‐based dosing and dosing frequency.

## Methods

### Clinical Trials

The population PK analysis was performed using tralokinumab serum concentration–time data from 10 clinical trials: 3 phase 3 trials,^16^, ^17^ 4 phase 2 trials, and 3 phase 1 trials across healthy subjects, subjects with asthma, and subjects with moderate to severe AD. The clinical trials included single or multiple doses of tralokinumab, ranging from 1.0 to 10.0 mg/kg administered intravenously (IV) or 150 to 600 mg administered SC every 2 or 4 weeks for up to 52 weeks. The trials are identified in Table S1, along with key design features including treatment groups and PK sampling details.

All the trials included were performed in accordance with Good Clinical Practice guidelines and adhered to the Declaration of Helsinki. All trial protocols were approved by the relevant health authorities and ethics committees, and written informed consent was obtained from each subject before any trial‐related procedure was initiated.

### Assay

Tralokinumab serum samples were analyzed using a validated sandwich immunoassay as previously described.^20^ The assay was updated and revalidated during the clinical development program, resulting in varying lower limits of quantification (LLOQs) across the trials: 0.3 μg/mL in trials CAT‐354‐0602, CAT‐354‐0703, and MI‐CP199, MI‐CP224; 0.5 μg/mL in trial CD‐RI‐CAT‐354‐1049; and 0.1 μg/mL in trials D2213C00001, ECZTRA 1, ECZTRA 2, ECZTRA 3, and ECZTRA 5. In the ECZTRA trials, the cumulative bias of quality control samples ranged between –4.8% and 1.2% and the cumulative precision was ≤31.5% (≤17.2% when statistical outliers were excluded). In D2213C00001, the cumulative bias of quality control samples ranged between –8.2% and –4.7% and the cumulative precision was ≤9.2%.

### Population PK Analysis

A nonlinear mixed effect modeling approach was used for the population PK analysis. The model was built from 3 components. The first, the fixed effects (or structural) model, described all the typical PK parameters, such as the volume of distribution or clearance for the average subject. The second component, the random effects (or statistical) model, described all the unexplained variability, including interindividual, interoccasion, and intraindividual (or residual error) variability in terms of variance estimates. The third component, a covariate model, was implemented to account for other intrinsic or extrinsic factors that may explain the variability in PK, such as age, body weight, or race.

A 2‐compartment model with first‐order absorption and elimination was used as the initial structural model; the PK of tralokinumab appears to be linear and biphasic after IV administration. The model was parameterized in terms of an absorption rate constant (k_a_), central and peripheral volumes of distribution (V_2_ and V_3_, respectively), and clearance (CL) and intercompartmental clearance . As the dataset contained data from both IV and SC administration, the absolute bioavailability (F) of the SC formulation was estimated.

In the statistical model, interindividual variability (IIV) was included as multiplicative exponential terms (assuming log‐normal IIV) on selected model parameters. The residual unexplained variability was accounted for using a combined additive and proportional residual error model. An off‐diagonal term was included in the covariance matrix to account for the correlation between CL and V_2_.

Other structural and statistical models were also evaluated, including evaluation of (1) a 1‐compartment model; (2) log‐normal IIV on all volume and clearance parameters; and (3) additive, proportional, and combined error models. Additionally, the covariate body weight (allometric scaling) was evaluated on volume and clearance parameters when selecting the structural model, as body weight has been an important covariate for most antibodies.^21^

Body weight, height, body mass index, and lean body mass are correlated. Of these, only body weight was evaluated as a covariate, in line with the general approach for population PK analyses of antibodies.^22^ Other demographic factors tested in the covariate model were age, sex, race, and ethnicity. Markers of renal and hepatic impairment were tested as disease‐related covariates, along with disease type (healthy, asthma, or AD) and disease severity (baseline Eczema Area and Severity Index [EASI] score). Another covariate tested was “ECZTRA trials” (ie, 1 phase 2 and 3 phase 3 trials in AD with the trial acronym ECZTRA) versus “non‐ECZTRA” trials (ie, other trials, including 1 phase 2b trial in AD). Furthermore, dilution vs no dilution of dose before SC administration was evaluated as a trial‐related covariate. The rationale for including this covariate was that 150 mg/mL tralokinumab was injected undiluted in all trials with SC administration except for in the 45 mg/mL dose group of the phase 2b trial D2213C00001, where 0.3 mL of the 150 mg/mL solution was diluted to 1 mL for the purpose of blinding. In this regard, it was observed in an earlier analysis that the exposure in subjects dosed with tralokinumab at a concentration of 45 mg/mL was higher, relative to the dose level, than in subjects dosed at 150 mg/mL.

The covariate‐parameter relationships were implemented as power models for continuous covariates and as fractional change parameters for categorical covariates. For a covariate *x*, these 2 covariate models can be illustrated as:

$$\text{Continuous}\;\text{covariate:}\;ParCov_{x}={\left(Cov/Cov_{ref}\right)}^{\theta x}$$

$$\text{Categorical}\;\text{covariate:}\;ParCov_{x}=\left(1+\theta_{xn}\right)\;\mathrm{if}\;Cov=Cov_{catn}$$

where *Cov_ref_* is the reference covariate value used for normalization, θ*_x_* and θ*_xn_* are the covariate‐parameters to be estimated, and *Cov_cat1_*, …, *Cov_catn_* are the possible *n* covariate categories.

The total effect of covariates on parameter *P* is then the product of the *m* covariate terms:

$$\mathrm{TY}P_{i}=P_{\mathrm{pop}}*\mathrm{ParCo}v_{1}*\mathrm{ParCo}v_{2}*\text{…}*\mathrm{ParCo}v_{m}$$

where *TVP_i_* is the typical value of parameter *P* for subject *i*, and *P_pop_* is the typical parameter value for a subject with the reference covariate value(s).

During the covariate model building, the impact of the covariates on PK parameters was investigated using an automated stepwise covariate modeling approach. In the forward steps, the subset of covariates that were potential predictors in the model were identified. In the backward steps, the identified covariates were reevaluated and eliminated if not statistically significant. A significance level of *P* < .01 was used for the forward inclusion criteria and *P* > .001 for the backward deletion criteria. Subsequently, covariates found to be statistically significant during the stepwise covariate modeling were evaluated as clinically relevant and included in the final model if they met the following criteria: for categorical covariates, an estimated absolute value of θ_xn_ >0.2; for continuous covariates,^23^ a ratio of >1.4 for the maximum and minimum effect of the covariate (calculated by inserting the minimum and maximum values into the above expression for covariate‐parameter relationships).

Model selection was based on comparison of the objective function value (OFV) between hierarchical models, goodness‐of‐fit diagnostic plots, visual predictive check (VPC) plots,^24^ as well as physiological plausibility and precision of model parameter estimates. For comparison of 2 hierarchical models, approximate *P* values were derived from the change in OFV using the χ^2^ distribution. For inclusion of a parameter in the model, a covariate or variance was considered significant if the OFV decreased with at least 6.64 points, equivalent to *P* < .01 for 1 degree of freedom.

To construct the VPC, data from subjects with AD receiving 300 mg every 2 weeks were extracted from the analysis data set. One thousand replicates of the extracted data set were simulated and 95%CIs for the 5th, 50th, and 95th percentiles of the simulated serum concentrations were calculated and plotted together with the 5th, 50th, and 95th percentiles of the observed serum concentrations. Simulated concentration values below the LLOQ were excluded.

Tralokinumab population PK characteristics such as area under the concentration‐time curve (AUC) in a dosing interval at steady state, elimination half‐life, and accumulation ratio for an every‐2‐week dosing schedule were derived using model‐based simulations with the final model. The impact of any identified clinically significant covariates, as well as dosing frequency, was also investigated using model simulations.

The mixed‐effect analysis was performed using NONMEM 7.4 (ICON Development Solutions, Dublin, Ireland). The NONMEM routine ADVAN 13 was used for parameter estimation. Model simulations were performed within NONMEM and graphical presentation of results was done using R version 3.4.2/RStudio version 1.1.383 (R Foundation for Statistical Computing, Vienna, Austria).

## Results

### Data Set for Population PK Analysis

The analysis set was defined as all subjects who received at least 1 dose of tralokinumab and had at least 1 quantifiable serum concentration of tralokinumab. A total of 490 serum concentrations taken after dosing of tralokinumab were below LLOQ and were excluded from the analysis. Accordingly, the final data set included 13 361 quantifiable serum concentrations of tralokinumab from 2561 subjects. The key characteristics of the population are shown in Table 1.

**Table 1:** Key Characteristics of the 2561 Subjects Included in the Final Data Set

	**Median (Range)**
Age, y^a^	38 (18‐92)
Weight, kg	74.5 (36‐165)
Eczema Area and Severity Index score at baseline^b^	27.5 (12‐72)
Estimated glomerular filtration rate, mL/min^c^	106 (28‐154)
	**Number (percentage) of subjects**
Male/Female sex	1410 (55)/1151 (45)
White/Asian/Black or African American	1721 (67)/560 (22)/183 (7)
Not Hispanic or Latino/Hispanic or Latino	2339 (91)/222 (8.7)
Hepatic impairment: none/mild/moderate or severe^d^	2116 (83)/436 (17)/9 (0.4)
Atopic dermatitis/asthma/healthy	2066 (81)/441 (17)/2066 (2)
ECZTRA (ECZema TRAlokinumab) trial^e^	2204 (86)
No dilution of dose^f^	2512 (98)

^a^ 131 subjects were ≥65 years.

^b^ Only for subjects with atopic dermatitis.

^c^ 1 subject had <30 mL/min, 47 subjects had 30‐59 mL/min, and 522 subjects had 60‐89 mL/min, corresponding to severe, moderate, and mild renal impairment, respectively, according to the National Kidney Foundation classification^29^ and based on values calculated using the Chronic Kidney Disease Epidemiology Collaboration equation.^30^

^d^ None: bilirubin, aspartate aminotransferase (AST), and alanine aminotransferase (ALN) below the upper limit of normal (ULN); mild: bilirubin between ULN and 1.5 × ULN, or AST or ALT > ULN; moderate or severe: bilirubin > 1.5 × ULN, according to the National Cancer Institute Organ Dysfunction Working Group criteria for hepatic dysfunction.^31^

^e^ Key phase 2 and phase 3 trials in subjects with atopic dermatitis.

^f^ 150 mg/mL tralokinumab was injected undiluted in all trials with subcutaneous administration except for in the 45‐mg/mL dose group of trial D2213C00001, where 0.3 mL of the 150‐mg/mL solution was diluted to 1 mL for the purpose of blinding.

The values used for normalization of continuous covariates in the covariate analysis were age, 35 years; body weight, 75 kg; baseline EASI score, 25 (only for subjects with AD); and estimated glomerular filtration rate (eGFR), 100 mL/min.

Only the 3 largest race groups (White, Asian, and Black or African American; see Table 1) were considered large enough to be included in the covariate analysis. The remaining race groups (American Indian or Alaska Native, Native Hawaiian or Other Pacific Islander, and other/multiple, accounting for 4% of subjects) were pooled with the White group.

### Final Model

**Figure 1:**
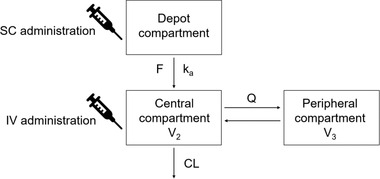
Structural representation of the 2‐compartment population pharmacokinetic model of tralokinumab, including first‐order absorption and linear elimination. CL, clearance; F, bioavailability; IV, intravenous; k_a_, absorption rate constant; Q, intercompartmental clearance; SC, subcutaneous; V_2_, central volume of distribution; V_3_, peripheral volume of distribution.

The base model constituted a 2‐compartment model with first‐order absorption and elimination from the central compartment with log‐normal IIV on V_2_ and CL, a combined additive and proportional error model, and common allometric exponents implemented for volume and CL parameters (1 exponent for volume parameters and 1 for clearance parameters). Other combinations of allometric exponents on CL and volume parameters were investigated for up to 4 parameters; the one implemented had the lowest OFV in combination with the fewest parameters. The structural model of the base model is illustrated in Figure 1.

During the stepwise covariate search, the following covariates, in addition to body weight, were found to have a statistically significant effect on model parameters: non‐ECZTRA trials, age, baseline EASI score, eGFR, Hispanic or Latino, Asian, Black or African American, female sex, and asthma had an effect on CL; non‐ECZTRA trials, age, Asian, Black or African American, and female sex had an effect on V_2_; dilution had an effect on F and k_a_.

Based on the criteria for clinical relevance described under Methods, the following covariates were found to have a clinically relevant effect: non‐ECZTRA trials on V_2_ and CL; dilution on F and k_a_; and body weight on V_2_, V_3_, CL, and Q, as included earlier in the model evaluation. The final model parameters are shown in Table 2. The covariate non‐ECZTRA trials on CL was estimated to 0.344. Since CL is the only parameter affecting steady state AUC, a 34.4% higher clearance corresponds to a 25.6% lower AUC at steady state. The estimated allometric exponents for volume (0.783) and clearance (0.873) parameters are comparable to what has previously been described for other monoclonal antibodies.^22^ For a power model of weight on clearance with an exponent of 0.873, a 2.21 fold change in weight leads to a 2‐fold change in clearance or AUC. Compared to a 75‐kg subject, a body weight <33.9 kg or >166 kg will result in a 2‐fold increase or decrease of AUC.

**Table 2:** Final Model: Population PK Parameter Estimates

**Parameter**	**Unit**	**Estimate** **^a^**	**RSE (%)** **^b^**	**95%CI** **^c^**	**Shrinkage (%)**
**PK parameter**					
k_a_ (absorption rate constant)	day	0.184	4	0.164 to 0.202	…
V_2_ (central volume of distribution)	L	2.71	7	2.33 to 3.00	…
CL (clearance)	L/day	0.149	5	0.136 to 0.164	…
V_3_ (peripheral volume of distribution)	L	1.44	6	1.21 to 1.71	…
Q (intercompartmental clearance)	L/day	0.159	8	0.125 to 0.199	…
F (bioavailability)	Unitless	0.761	5	0.697 to 0.831	…
σ additive	μg/mL	0.238	18	0.123 to 0.366	…
σ proportional	CV	0.216	1	0.207 to 0.224	…
**IIV** **^d^**					
IIV on V_2_	CV%	40.1	4	34.4 to 48.5	27
IIV on CL	CV%	31.3	2	29.6 to 33.0	7
IIV on V_2_:CL	Corr.^e^	0.61	…	…	…
**Covariate**					
V_2_ and V_3_ ∼ weight	Unitless	0.783	4	0.727 to 0.842	…
CL and Q ∼ weight	Unitless	0.873	3	0.816 to 0.930	…
CL ∼ non‐ECZTRA trials	Unitless	0.344	6	0.308 to 0.387	…
V_2_ ∼ non‐ECZTRA trials	Unitless	0.258	12	0.198 to 0.327	…
F ∼ dilution^f^	Unitless	0.354	19	0.220 to 0.499	…
k_a_ ∼ dilution^f^	Unitless	–0.519	9	–0.605 to 0.394	…

IIV, interindividual variability; PK, pharmacokinetic; RSE, relative standard error.

^a^ Covariates were implemented as exemplified for clearance for non‐ECZTRA trials: CL = 0.149 L/h × (body weight/75 kg)^0.873^ × (1 + 0.344), using power models for continuous covariates and fractional change for categorical covariates.

^b^ RSE was obtained from the COVARIANCE option in NONMEM.

^c^ 95%CI was obtained from bootstrap (1000 samples and stratified on STUDY).

^d^ IIV (inter‐individual variability) was calculated as √$\left(e^{\omega^{2}}-1\right)$.

^e^ Correlation (Corr.) was calculated as $\rho_{i,j}=\frac{\omega_{i,j}^{2}}{\omega_{i,i}\cdot \omega_{j,j}}$.

^f^ In the 45‐mg dose group of trial D2213C00001, tralokinumab was diluted before subcutaneous administration. In all other trials with subcutaneous administration, tralokinumab was injected undiluted.

The IIV, implemented using the OMEGA BLOCK option in NONMEM (correlation = 61%) and expressed as percent coefficient of variation, was estimated to be 40.1% for V_2_ and 31.3% for CL. The degree of shrinkage for IIV on V_2_ (27%) and CL (7%) was acceptable (<30%). In the nonparametric bootstrap, 98% of the runs converged, indicating good model stability.

**Figure 2:**
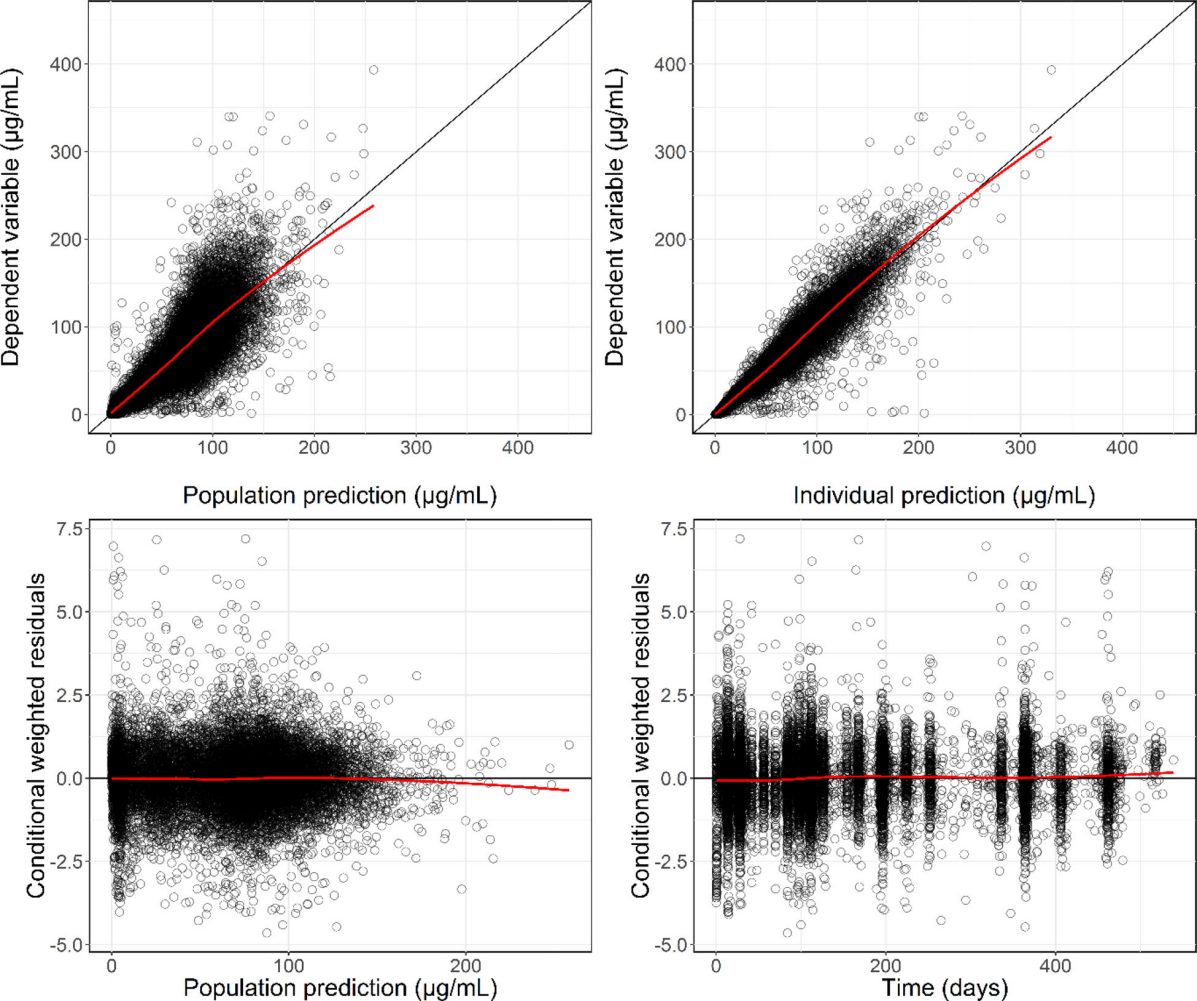
Final model: goodness‐of‐fit plots. **Top left**: Correlation between the dependent variable (tralokinumab serum concentration) and the population predictions. **Top right**: Correlation between the dependent variable (tralokinumab serum concentration) and the individual predictions. **Bottom left**: Correlation between the conditional weighted residuals and the population predictions. **Bottom right**: Correlation between the conditional weighted residuals and time. The black circles represent the individual observations/predictions/conditional weighted residuals and the red line is the trend line (locally estimated scatterplot smoothing). The black line in the upper panels is the line of unity.

To evaluate whether the model was able to describe the observed data, a series of goodness‐of‐fit plots was generated (Figure 2). As evident from the figure (top left and right), the observed vs population‐ and individually predicted concentrations showed a random normal scatter around the identity, indicating no major systematic bias of the model. A similar trend was observed for the conditional weighted residuals vs population prediction (bottom left part of the figure). Finally, no time‐dependent bias was observed for the conditional weighted residuals vs time, suggesting absence of time‐dependent PK (bottom right).

**Figure 3:**
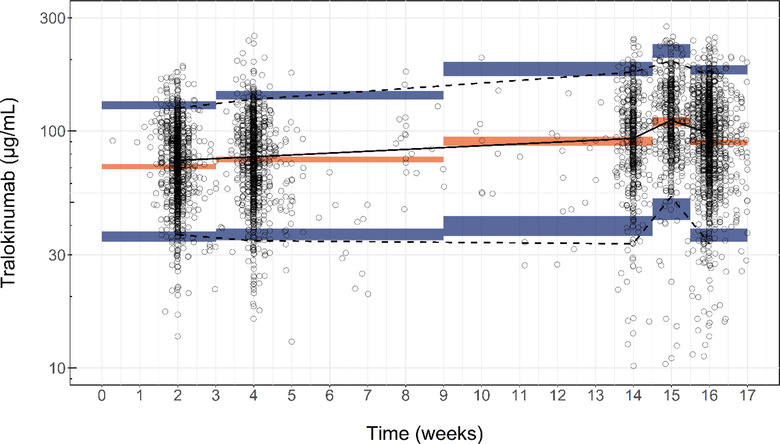
Final model: visual predictive check for weeks 0‐16 in ECZTRA trials (subjects with atopic dermatitis). Visual predictive check (VPC) of the serum concentration‐time profile of tralokinumab in subjects with atopic dermatitis following subcutaneous administration of tralokinumab 300 mg (including loading dose), depicting the observed concentration of tralokinumab (circles), the median of the observed concentration of tralokinumab (solid line), the 95%CI of the simulated median (orange shaded area), the 95%CI of the simulated lower 5th and the upper 95th percentiles (blue shaded areas), and the observed 5th and 95th percentile (dashed line). For visual purposes, serum concentrations <10 μg/mL were excluded from the plot (not for the calculation of VPC statistics).

To assess how well the final model was able to describe the observed data, 3 VPCs were generated (as also done for the base model). As apparent from the VPC of the serum concentration–time profile for the ECZTRA trials (Figure 3), the final model provided an adequate description of the observed data: The observed median as well as the observed lower and upper percentiles were captured by the 95%CI of the simulated median and lower and upper percentiles. However, the final model slightly underpredicted the observed median at time points before 3 weeks and after 15.5 weeks. VPCs for 2 non‐ECZTRA trials are included in the Supplemental Information, 1 trial in healthy subjects and 1 trial in subjects with AD. For both single IV dosing of 150 mg and single subcutaneous dosing of either 150 or 300 mg in healthy subjects and multiple dosing of 45, 150, and 300 mg in subjects with AD, the observed data are well described by the 95%CIs of the simulated median, lower, and upper percentiles. The 95%CIs are wider for the single‐dose trial, as there are fewer subjects in that trial.

No major correlations between η values (interindividual random effect) for V_2_ and CL and covariates were identified for the final model (data not shown). This indicates that the model adequately accounts for all important parameter‐covariate relationships.

### Evaluation of the Impact of Body Weight on Exposure

**Figure 4:**
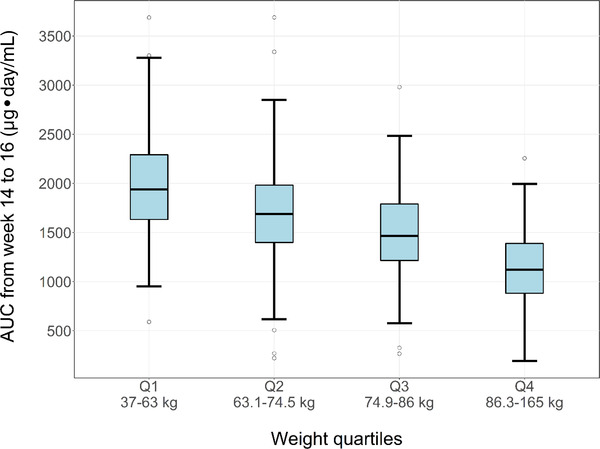
Final model: area under the serum concentration‐time curve (AUC) from week 14 to 16 vs weight quartiles in ECZTRA trials. Boxplot depicting the correlation between individually predicted AUC from week 14 to 16 for all subjects in ECZTRA trials, grouped by weight quartiles (approximative quartiles with n = 361 in Q1, n = 356 in Q2, n = 363 in Q3, n = 350 in Q4). The top, middle, and bottom of each box are the third quartile, median, and the first quartile of data in each category. The whiskers are drawn to the nearest value not beyond 1.5 times the interquartile range (IQR). The circles represent individual values outside 1.5 times the IQR. Only data from subjects who received ≥6 doses of tralokinumab during the first 119 days and who had a PK sample in the time interval 105 to119 days are included in the plot.

To investigate the influence of body weight on exposure, the model‐predicted AUC from week 14 to 16 was computed for each subject, and the subjects were divided into quartiles based on body weight. There was a clear correlation between AUC from week 14 to 16 and body weight (Figure 4). However, it was also evident that body weight accounts for only a small amount of the overall variability seen for exposure: <2‐fold difference between the medians of quartile 1 (1941 μg × day/mL) and quartile 4 (1125 μg × day/mL). Furthermore, the ranges of individually predicted AUCs across the different weight quartiles are overlapping.

**Figure 5:**
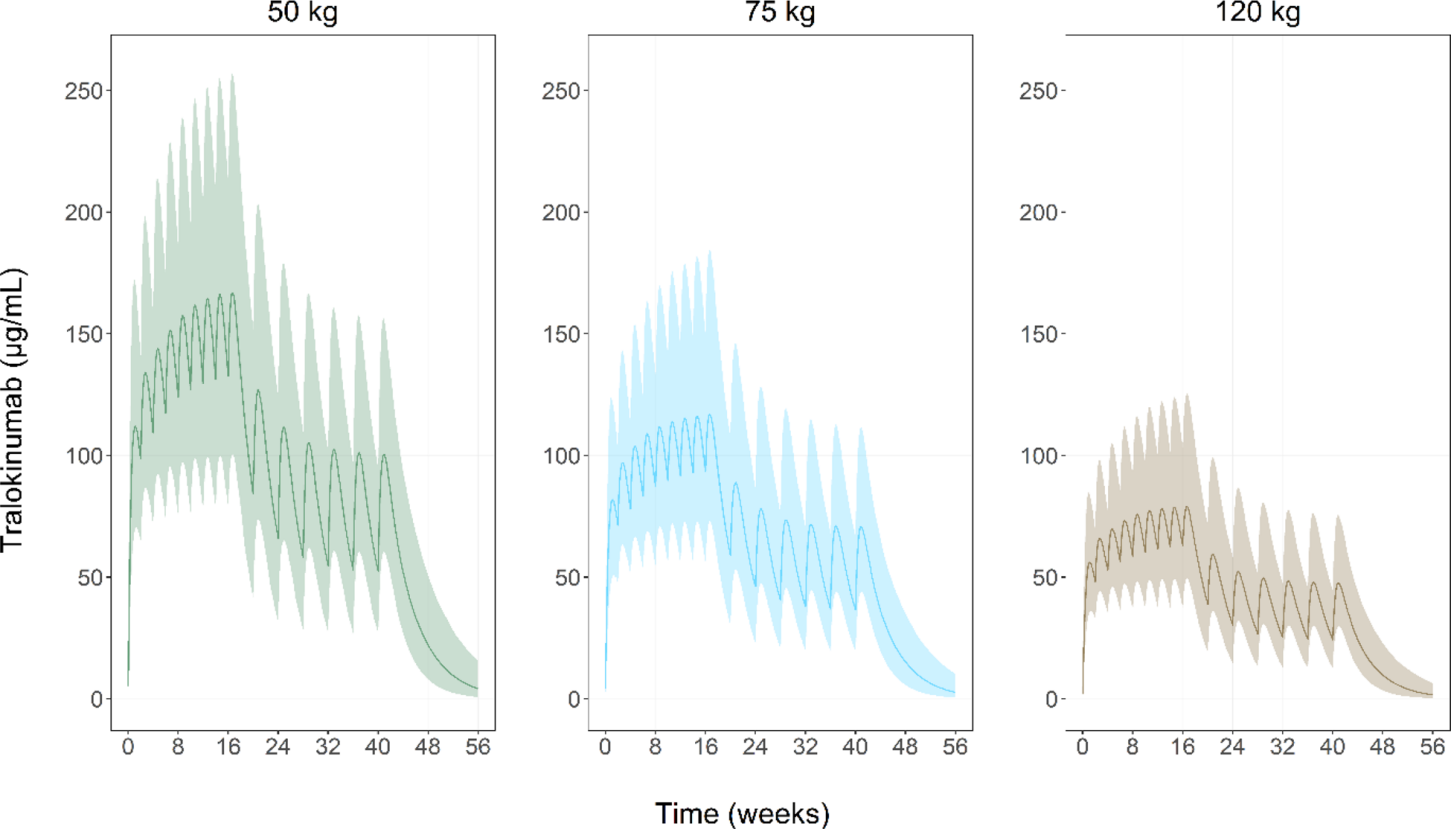
Final model simulations. Serum concentration‐time profile for tralokinumab, simulated using the final population pharmacokinetic model and based on the following dosing scenario: an initial loading dose of 600 mg at week 0, 300 mg every 2 weeks from week 0 to week 14, and a maintenance dose of 300 mg every 4 weeks from week 16 to week 40 (steady state) for a subject with a body weight of either 50, 75, or 120 kg. For each dose 1000 subjects were simulated and the plots show the 5th, 50th, and 95th percentiles in the population.

To further elucidate the relationship between body weight, exposure, and dosing regimen, concentration‐time profiles were simulated for 3 typical subjects with different body weight, each receiving a loading dose of 600 mg of tralokinumab at baseline followed by 300 mg every 2 weeks for 16 weeks and 300 mg every 4 weeks thereafter (Figure 5). The figure shows a large overlap of simulated serum concentrations–time profiles for the 75‐kg subject with both the 50‐kg subject and the 120‐kg subject. Since body weight affects both CL and volume of distribution, changes in body weight affect not only the average serum concentration but also the difference between minimum and maximum concentrations at steady state.

**Figure 6:**
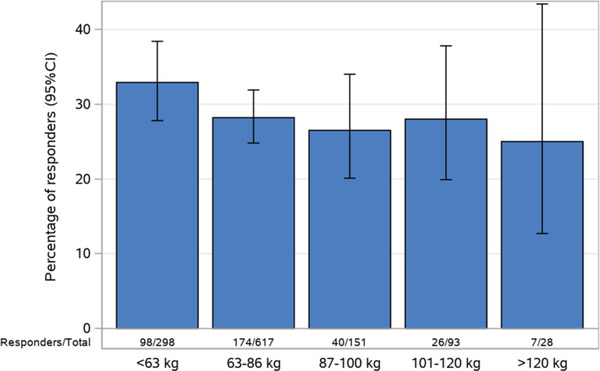
Percentage of EASI‐75 responders by body weight subgroups in ECZTRA 1 and ECZTRA 2 trials. Percentage of responders, defined as at least 75% reduction in Eczema Area and Severity Index (EASI‐75) from baseline to week 16, by body weight subgroups in the clinical trials ECZTRA 1 and ECZTRA 2. All subjects treated with tralokinumab in the initial part of each trial received a dose of 300 mg every 2 weeks. Subjects who received rescue medication were considered nonresponders, and subjects with missing data at week 16 were imputed as nonresponders. Body weight was split into groups by the 25th and 75th percentiles, and the highest body weight group was further split into 101 to 120 kg and >120 kg.

## Discussion

Population PK modeling was used to integrate tralokinumab PK data obtained during drug development for the purpose of quantitatively characterizing the PK of tralokinumab and identifying important predictors of tralokinumab exposure. The final population PK model, a 2‐compartment model with linear absorption and elimination kinetics, provided an adequate description of the observed concentration‐time profile of tralokinumab in healthy subjects, subjects with asthma, and subjects with AD. As the population PK model was based on a comprehensive data set, including a wide range of covariates and doses as well as both IV and SC dosing, it is expected that robust and reliable conclusions regarding tralokinumab PK may be derived from the model.

Dose‐proportional PK was observed for all dose levels of tralokinumab, suggesting no influence of target‐mediated drug disposition. This is in line with previous observations regarding antibody PK, suggesting that antibodies directed against soluble targets, such as tralokinumab, often display linear PK.^25^ Since the PK of tralokinumab appeared to be linear and the elimination phase was adequately captured, the values below LLOQ were not included in the analysis.

The volume of distribution at steady state, calculated based on the final population PK model, was estimated to be 4.15 L (V_2_ + V_3_ in Table 2). The estimated CL (0.149 L/day), SC bioavailability (76.1%), absorption rate constant (0.184 day) and t_½_ (22.0 days) were all in close agreement with previously estimated PK parameters for tralokinumab^19^ and other monoclonal antibodies.^22^, ^26^, ^27^ Accumulation of tralokinumab following multiple dosing was predicted to be 3‐fold without a loading dose and 1.5‐fold with a loading dose of 600 mg of tralokinumab.

Therapeutic monoclonal antibodies have the potential to induce an immunological response leading to generation of antidrug antibodies. Potential clinical consequences of immunogenicity range from little or no effect to altered PK, reduced efficacy, and safety issues such as induction of anaphylaxis/hypersensitivity reactions, immune‐complex disease, and serum sickness.^28^ Therefore, all clinical trials with tralokinumab included testing of serum samples for antidrug antibodies, and if positive also for neutralizing antibodies. The observed incidence was low (the incidence of ADA up to 16 weeks was 1.4% for patients treated with tralokinumab and 1.3% for patients treated with placebo), and the presence of neutralizing antibodies was not deemed to have an impact on the efficacy or safety of tralokinumab.^16^ Hence, antidrug antibodies were not included as a covariate in the population PK analysis.

Body weight, non‐ECZTRA trials, and concentration of the drug formulation were deemed clinically relevant covariates, based on the criteria described under Methods. The covariate “non‐ECZTRA trials” accounts for the observation that higher exposures were seen in the recent ECZTRA trials than in previous trials. The PK data included in the analysis originate from trials conducted over a 14‐year period by different pharmaceutical companies using different bioanalytical laboratories. By including only 1 covariate to account for all these sources of variability, it was possible to develop a model providing a satisfactory description of data from all trials included. The covariate “dilution of dose” accounts for the observation that the 45‐mg dose used in the phase 2b trial (D2213C00001) was diluted to maintain the blind in the trial. All other SC administrations were undiluted. The dilution of the formulation appeared to affect the bioavailability, giving higher exposure than anticipated for this dose. As non‐ECZTRA trials and concentration of the drug formulation (dilution) are extrinsic factors related to the drug development process, these covariates do not have any relevance for the future clinical use of the tralokinumab 150‐mg/mL solution. Thus, body weight was ultimately the only clinically meaningful covariate among those evaluated.

When the impact of body weight on exposure, expressed as individually predicted AUC from week 14 to 16, was evaluated for all subjects in the ECZTRA trials, a less than 2‐fold difference in exposure was observed between the upper‐ and lower‐weight quartiles. In view of this relatively small difference, along with the overlapping ranges of individually predicted AUCs across the different weight quartiles, weight‐based dosing is not considered warranted. This evaluation is supported by the small differences in efficacy response identified between different weight subgroups of subjects with AD in the EZCTRA 1 and 2 trials (Figure 6).

Given the reduced exposure in patients with higher body weight, coupled with the reduced exposure provided by every‐4‐week dosing, it is uncertain whether patients with higher body weight will achieve sufficient exposure to maintain efficacy with the every‐4‐week dosing regimen. This is reflected in the tralokinumab product information, which states that reducing the dosage to every fourth week might not be appropriate for patients with a body weight >100 kg who achieve clear or almost clear skin after 16 weeks of treatment. This recommendation, which is considered at the discretion of the prescribing physician, is based on the analysis of exposure. A limited number of subjects received every‐4‐week maintenance dosing in the phase 3 trials, and therefore a reliable relationship between body weight and efficacy for every‐4‐week dosing could not be established.

To investigate the impact of body weight on the PK of tralokinumab in younger populations with AD, the population PK model reported here will be updated when data from an ongoing trial in adolescents become available (NCT03526861). Later, the model will also be updated with data from planned trials in younger children with AD. Such updates of the model with data from younger subjects with lower body weight will support decisions on the most appropriate dosing regimens, including consideration of weight‐based dosing, in pediatric populations.

## Conclusion

A 2‐compartment model with first‐order absorption (SC) and elimination adequately described the concentration‐time profiles of tralokinumab administered IV or SC to adult subjects with AD, asthma, or healthy subjects during phase 1, 2, and 3 clinical trials. Body weight had a significant impact on CL and volume of distribution of tralokinumab. Of the other covariates tested (age, sex, race, ethnicity, eGFR, hepatic impairment, baseline EASI score, and disease type), none were identified as clinically relevant predictors of tralokinumab PK. For body weight, the difference in exposure between the upper‐ and lower‐weight quartiles in patients with AD was <2‐fold, supporting the appropriateness of flat dosing (300 mg). Given the reduced exposure associated with higher body weight, coupled with the reduced exposure provided by every‐4‐week dosing, it is uncertain whether high‐weight patients will achieve sufficient exposure to maintain efficacy with the every‐4‐week dosing regimen instead of the standard every‐2‐week regimen. This is based on the analysis of exposure, as a reliable relationship between body weight and efficacy for every‐4‐week dosing could not be established due to a limited number of subjects receiving every‐4‐week maintenance dosing in the phase 3 trials.

This population PK model will serve as a useful tool to further investigate the exposure‐effect relationship of tralokinumab and the impact of body weight in different populations with moderate to severe AD.

## Conflicts of Interest

The authors declare no conflicts of interest.

## Supporting information

Supporting Information
