# Supplementary material for: Population Pharmacokinetics of Tralokinumab in Adult Subjects With Moderate to Severe Atopic Dermatitis
Source: Clin Pharmacol Drug Dev. 2022 Jun 7;11(8):910–21. doi: 10.1002/cpdd.1113 (PMC9796478; doi:10.1002/cpdd.1113)
Supplement: Supplementary file 1 — Supporting Information [file CPDD-11-910-s001.docx]

Table S1 Overview of clinical trials included in population pharmacokinetic analysis

| Trial ID, NCT number | Phase, type, duration | Population | Dose, route, regimen (n=exposed subjects) | PK sampling times, number of samples^a^ |
| --- | --- | --- | --- | --- |
| **Phase 1 and 2 trials in healthy subjects and subjects with asthma** | | | | |
| CAT-354-0703  NCT00638989 | Phase 1  Single dose | Healthy adult subjects | Tralokinumab:   - 150 mg, IV (n=10) - 150 mg, SC (n=10) - 300 mg, SC (n=10) | 16 time points: end-of-infusion, 0.5, 1, 3, 8 hours and Days 1, 3, 5, 7, 9, 14, 21, 28, 35, 42, 56  (S=503, S_Q_=398) |
| MI-CP224  NCT01093040 | Phase 1  Single dose | Healthy adult subjects | Tralokinumab, SC:   - 150 mg (n=8) - 300 mg (n=8) - 600 mg (n=8)   Placebo, SC (n=6) | 16 time points: 0.5, 1, 3, 8 hours and Days 1, 3, 5, 7, 9, 14, 21, 28, 35, 42, 56, 70  (S=408, S_Q_=319) |
| CAT-354-0602  NCT00974675 | Phase 1  Multiple dose  12 weeks (3 doses) | Adult subjects with asthma | Tralokinumab, IV, Q4W:   - 1.0 mg/kg (n=8) - 5.0 mg/kg (n=8) - 10.0 mg/kg (n=3)   Placebo, IV, Q4W (n=4) | 17 time points: 10 min, 12 hours and Days 4, 7, 14, 21, 28 (×3), 35, 56 (×3), 63, 84, 105, 147  (S=295, S_Q_=277) |
| MI-CP199  NCT00873860 | Phase 2a  Multiple dose  14 weeks (7 doses) | Adult subjects with asthma | Tralokinumab, SC, Q2W:   - 150 mg (n=47) - 300 mg (n=51) - 600 mg (n=47)   Placebo, SC, Q2W (n=48) | 11 time points: Days 15, 29, 43, 57, 71, 85, 88, 92, 99, 127, 169  (S=1,640, S_Q_=1,498) |
| CD-RI-CAT-354-1049  NCT01402986 | Phase 2b  Multiple dose  52 weeks (26/16 doses) | Adult subjects with asthma | Cohort 1:   - Tralokinumab 300 mg, SC, Q2W 52 weeks (n=150) - Placebo, SC, Q2W, 52 weeks (n=76)   Cohort 2:   - Tralokinumab 300 mg, SC, Q2W/12 weeks + Q4W/40 weeks (n=151) - Placebo, SC, Q2W/12 weeks + Q4W/40 weeks (n=75) | 11 time points: Weeks 1, 5, 13, 19, 25, 37, 49, 53, 59, 67, 75  (S=2,995, S_Q_=2,416) |
| **Phase 2 and 3 trials in subjects with atopic dermatitis** | | | | |
| D2213C00001  NCT02347176 | Phase 2b  Multiple dose  12 weeks (6 doses) | Adult subjects with atopic dermatitis | Tralokinumab, SC, Q2W:   - 45 mg (n=50) - 150 mg (n=51) - 300 mg (n=52)   Placebo, SC, Q2W (n=51) | 4 time points: Weeks 2, 4, 12, 22  (S=721, S_Q_=553) |
| ECZTRA 1  NCT03131648 | Phase 3  Multiple dose  52 weeks (26 doses) | Adult subjects with atopic dermatitis | Initial treatment   - Tralokinumab 300 mg (600 mg loading), SC, Q2W (n=602) - Placebo, SC, Q2W (n=196)   Maintenance treatment  Tralokinumab responders^c^ re-randomised:   - Tralokinumab 300 mg, SC, Q2W (n=68) - Tralokinumab 300 mg, SC, Q4W (n=76) - Placebo, SC, Q2W (n=35)   Placebo responders^c^: placebo, SC, Q2W (n=29)  Non-responders^c^: open-label^d^ tralokinumab 300 mg, SC, Q2W (n=563) | 8 time points: Weeks 2, 4, 14, 15, 16, 28, 52, 66^b^  (S=4,175, S_Q_=3,995) |
| ECZTRA 2  NCT03160885 | Phase 3  Multiple dose  52 weeks (26 doses) | Adult subjects with atopic dermatitis | Initial treatment   - Tralokinumab 300 mg (600 mg loading), SC, Q2W (n=592) - Placebo, SC, Q2W (n=200)   Maintenance treatment  Tralokinumab responders^c^ re-randomised:   - Tralokinumab 300 mg, SC, Q2W (n=91) - Tralokinumab 300 mg, SC, Q4W (n=89) - Placebo, SC, Q2W (n=46)   Placebo responders^c^: placebo, SC, Q2W (n=31)  Non-responders^c^: open-label^d^ tralokinumab 300 mg, SC, Q2W (n=558) | 7 time points: Weeks 2, 4, 14, 16, 28, 52, 66  (S=3,259, S_Q_=3,063) |
| **Phase 2 and 3 trials in subjects with atopic dermatitis (continued)** | | | | |
| ECZTRA 3  NCT03363854 | Phase 3  Multiple dose  32 weeks (16 doses) | Adult subjects with atopic dermatitis | Initial treatment   - Tralokinumab 300 mg (600 mg loading), SC, Q2W + TCS (n=252) - Placebo, SC, Q2W + TCS (n=126)   Continuation treatment  Tralokinumab responders^c^ re-randomised:   - Tralokinumab 300 mg, SC, Q2W + TCS (n=69) - Tralokinumab 300 mg, SC, Q4W + TCS (n=69)   Placebo responders^c^: placebo, SC, Q2W + TCS (n=41)  Non-responders^c^: tralokinumab 300 mg, SC, Q2W + TCS (n=174: 95 tralokinumab + 79 placebo non-responders) | 3 time points: Weeks 4, 16, 32  (S=906, S_Q_=820) |
| ECZTRA 5  NCT03562377 | Phase 2  Multiple dose  16 weeks (8 doses) | Adult subjects with atopic dermatitis | Tralokinumab 300 mg (600 mg loading), SC, Q2W (n=106)  Placebo, SC, Q2W (n=108) | 3 time points: Weeks 4, 16, 30  (S=213, S_Q_=212) |

**^a^** Scheduled time points after first dose of tralokinumab; S = total number of samples from all subjects, S_Q_ = total number of samples within limits of quantification.

**^b^** Japanese subjects in the open-label short-term extension arm had an additional sample at Week 68 and the final sample at Week 82 (that is, 9 time points).

**^c^** Responders in ECZTRA 1, 2 and 3 were defined as subjects with clinical response defined as IGA of 0 or 1, or at least 75% reduction in EASI score from baseline (EASI75) at Week 16.

**^d^** The open-label arm in ECZTRA 1 and 2 included subjects without clinical response^a^ at Week 16 from both tralokinumab and placebo groups. In addition, subjects in maintenance treatment who met certain criteria of non-responders could transfer to open-label treatment from Week 22. The open-label arm included optional TCS treatment.

**Abbreviations**: CTR = clinical trial report; EASI = Eczema Area and Severity Index; IGA = Investigator’s Global Assessment; IV = intravenous; PK = pharmacokinetic; Q2W = every 2 weeks; Q4W = every 4 weeks; SC = subcutaneous; TCS = topical corticosteroid

**Figure S1**


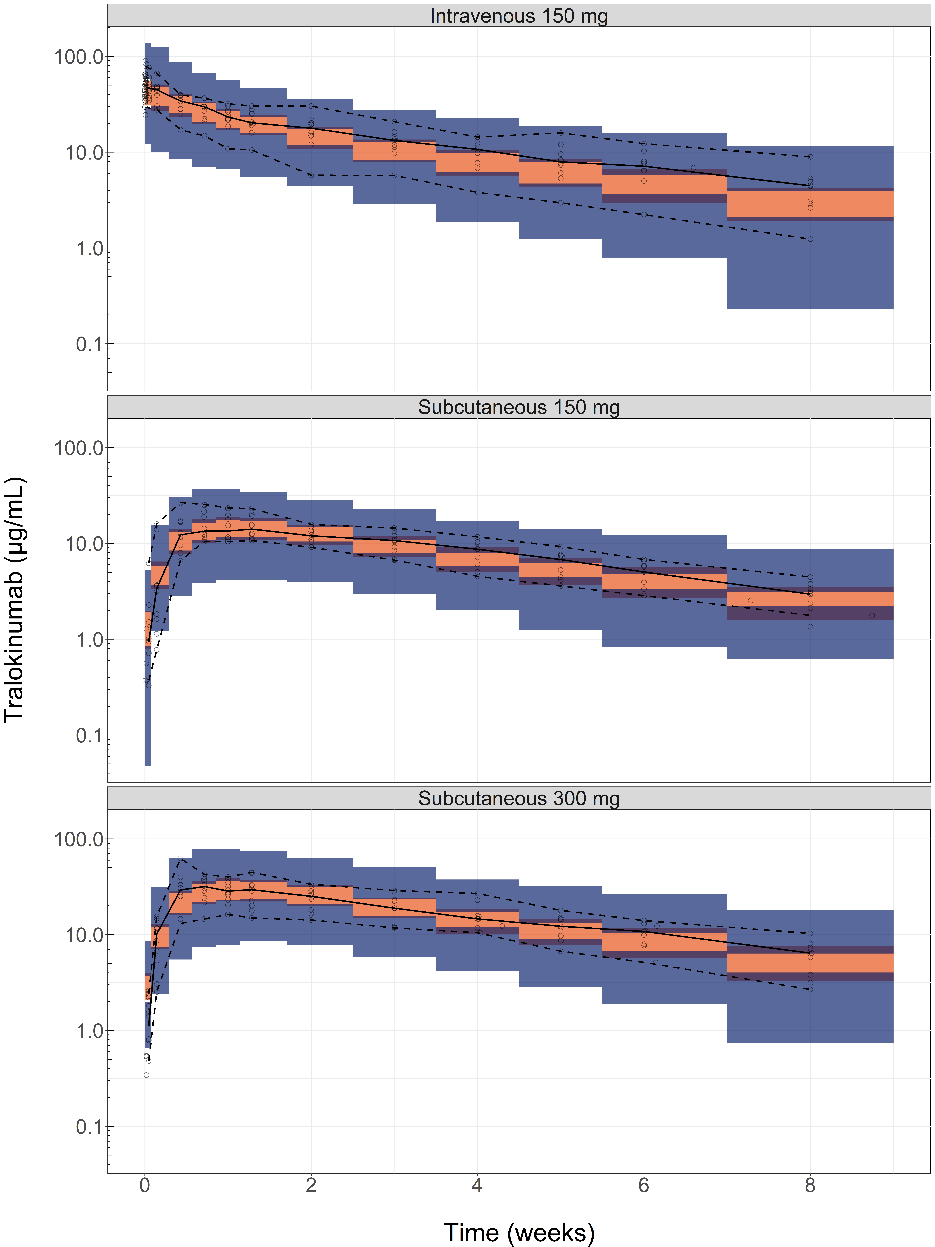


Visual predictive check (VPC) of the serum concentration–time profile of tralokinumab in healthy subjects following a single intravenous tralokinumab 150 mg or a single subcutaneous administration of either tralokinumab 150 or 300 mg (Trial CAT-354-0703), depicting the observed concentration of tralokinumab (circles), the median of the observed concentration of tralokinumab (solid line), the 95% confidence interval of the simulated median (orange shaded area), the 95% confidence interval of the simulated lower 5th and the upper 95th percentiles (blue shaded areas), and the observed 5th and 95th percentile (dashed line).

**Figure S2**


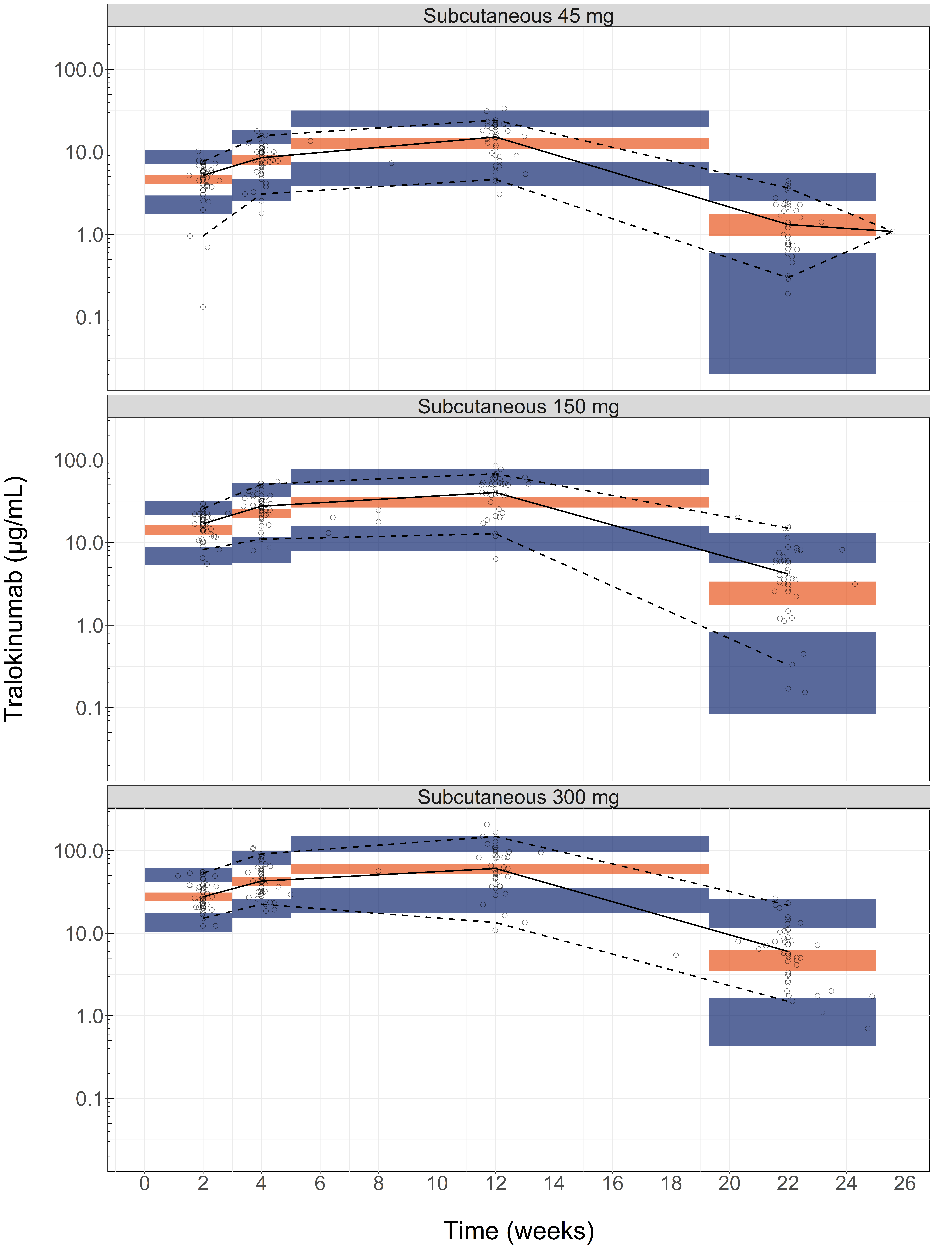


Visual predictive check (VPC) of the serum concentration–time profile of tralokinumab in subjects with atopic dermatitis following subcutaneous administration of either tralokinumab 45, 150 or 300 mg (Trial D2213C00001), depicting the observed concentration of tralokinumab (circles), the median of the observed concentration of tralokinumab (solid line), the 95% confidence interval of the simulated median (orange shaded area), the 95% confidence interval of the simulated lower 5th and the upper 95th percentiles (blue shaded areas), and the observed 5th and 95th percentile (dashed line).
